# First endemic freshwater *Gammarus* from Crete and its evolutionary history—an integrative taxonomy approach

**DOI:** 10.7717/peerj.4457

**Published:** 2018-03-09

**Authors:** Kamil Hupało, Tomasz Mamos, Weronika Wrzesińska, Michał Grabowski

**Affiliations:** Department of Invertebrate Zoology and Hydrobiology, University of Lodz, Lodz, Poland

**Keywords:** *Gammarus*, Crete, New species, Endemic, Integrative taxonomy, Evolutionary history, Multimarker approach, Amphipoda, Freshwater

## Abstract

The Mediterranean islands are known as natural laboratories of evolution with a high level of endemic biodiversity. However, most biodiversity assessments have focused mainly on terrestrial and marine fauna, leaving the freshwater animals aside. Crete is one of the largest islands in the Mediterranean Basin, with a long history of isolation from the continental mainland. Gammarid amphipods are often dominant in macrozoobenthic communities in European inland waters. They are widely used in biomonitoring and exotoxicological studies. Herein, we describe *Gammarus plaitisi* sp. nov., endemic to Cretan streams, based on morphological characters and a set of molecular species delimitation methods using mitochondrial cytochrome oxidase subunit I and 16S rRNA genes as well as nuclear 28S rDNA, ITS1 and EF1-alpha genes. The divergence of the new species is strongly connected with the geological history of the island supporting its continental origin.

## Introduction

Due to its complex geological history and unique combination of geological and climatic factors, the Mediterranean Region is recognized as one of the globally most important hotspots of biodiversity and endemism, and is a model system for studies of biogeography and evolution (Woodward, 2009; Poulakakis et al., 2015). The freshwater fauna of the region is still heavily understudied, yet it is estimated that the Mediterranean is inhabited by ca. 35% of Palearctic species, which mean the region contains more than 6% of the world’s freshwater species. At least 43% of the freshwater Mediterranean species are considered to be local endemics (Figueroa et al., 2013). Most of these endemics occupy the Mediterranean islands (Myers et al., 2000; Whittaker & Fernández-Palacios, 2007).

Crete is the fifth largest of the Mediterranean islands and the largest of the Aegean islands. At the beginning of the Miocene, Crete was a part of the mainland composed of the Balkan Peninsula and Asia Minor (23–12 million years ago). Around 12 million years ago, the split of the Balkan Peninsula (including Crete) from Asia Minor began. Afterwards, about 11–8 million years ago, the isolation of Crete from Peloponnesus started, due to the rise of sea levels. Later, between 5.96 and 5.33 million years ago, the dessication of the Proto-Mediterranean Sea during the Messinian Salinity Crysis led to the formation of hypersaline deserts around Crete and other islands, and this is the last known land connection between Crete and the mainland (Poulakakis et al., 2015). During the Pliocene, Crete was divided temporarily into at least four islands due to sea level rise associated with the Zanclean flood (Sondaar & Dermitzakis, 1982). At the end of the Pliocene or in the Early Pleistocene, Crete gained its present configuration.

Gammarid amphipods are among the most speciose, abundant and biomass-dominant, groups of benthic macroinvertebrates in lotic ecosystems in Europe and, particularly, in the Mediterranean Region (MacNeil, Dick & Elwood, 1997). They are also considered to be aquatic keystone species, structuring freshwater macroinvertebrate communities (Kelly, Dick & Montgomery, 2002). They are widely used as model organisms in biomonitoring and exotoxicological studies (i.e., Neuparth, Costa & Costa, 2002; Neuparth et al., 2005; Kunz, Kienle & Gerhardt, 2010). Gammarids are considered to be very good evolutionary models as they are exclusively aquatic organisms with limited dispersal abilities (Bilton, Freeland & Okamura, 2001). The majority of studies upon biodiversity of Mediterranean amphipods have focused exclusively on marine species, leaving the freshwater fauna relatively poorly known. So far, around 120 freshwater gammarid species living in the Mediterranean have been described, while only 15 species of two genera: *Gammarus* Fabricius, 1775 and *Echinogammarus* Stebbing, 1899, have been reported from the islands (Karaman & Pinkster, 1977a; Karaman & Pinkster, 1977b; Pinkster, 1993). Recently, an extraordinarily high rate of cryptic diversity was discovered within several morphospecies from both mentioned genera (Hou et al., 2011; Hou, Sket & Li, 2014; Weiss et al., 2014; Wysocka et al., 2014; Mamos et al., 2014; Mamos et al., 2016; Copilaş-Ciocianu & Petrusek, 2015; Copilaş-Ciocianu & Petrusek, 2017; Katouzian et al., 2016; Grabowski et al., 2017; Grabowski, Wysocka & Mamos, 2017). One can conclude that the number of species already reported from the Mediterranean islands is definitely underestimated. Moreover, molecular studies on insular species are absent. To date, there have been two freshwater endemic species reported from Crete, *E. kretensis* and *E. platvoeti*, both described by Pinkster (1993). Also, *Gammarus pulex pulex* (Linnaeus, 1758), a freshwater species widespread throughout Europe, has been reported from one locality on Crete (Karaman, 2003). No other insular freshwater *Gammarus* species has been reported from the Mediterranean.

In this paper, we show evidence that the Cretan population of *Gammarus pulex pulex* is, in fact, a new species and describe it as *Gammarus plaitisi* sp. nov., based on morphological, ultrastructural and molecular features. We also reconstruct, based on a multimarker dataset, the phylogeny of this species with respect to other lineages of *G. pulex* to reveal its biogeographic afiliations and possible origin.

**Figure 1:**
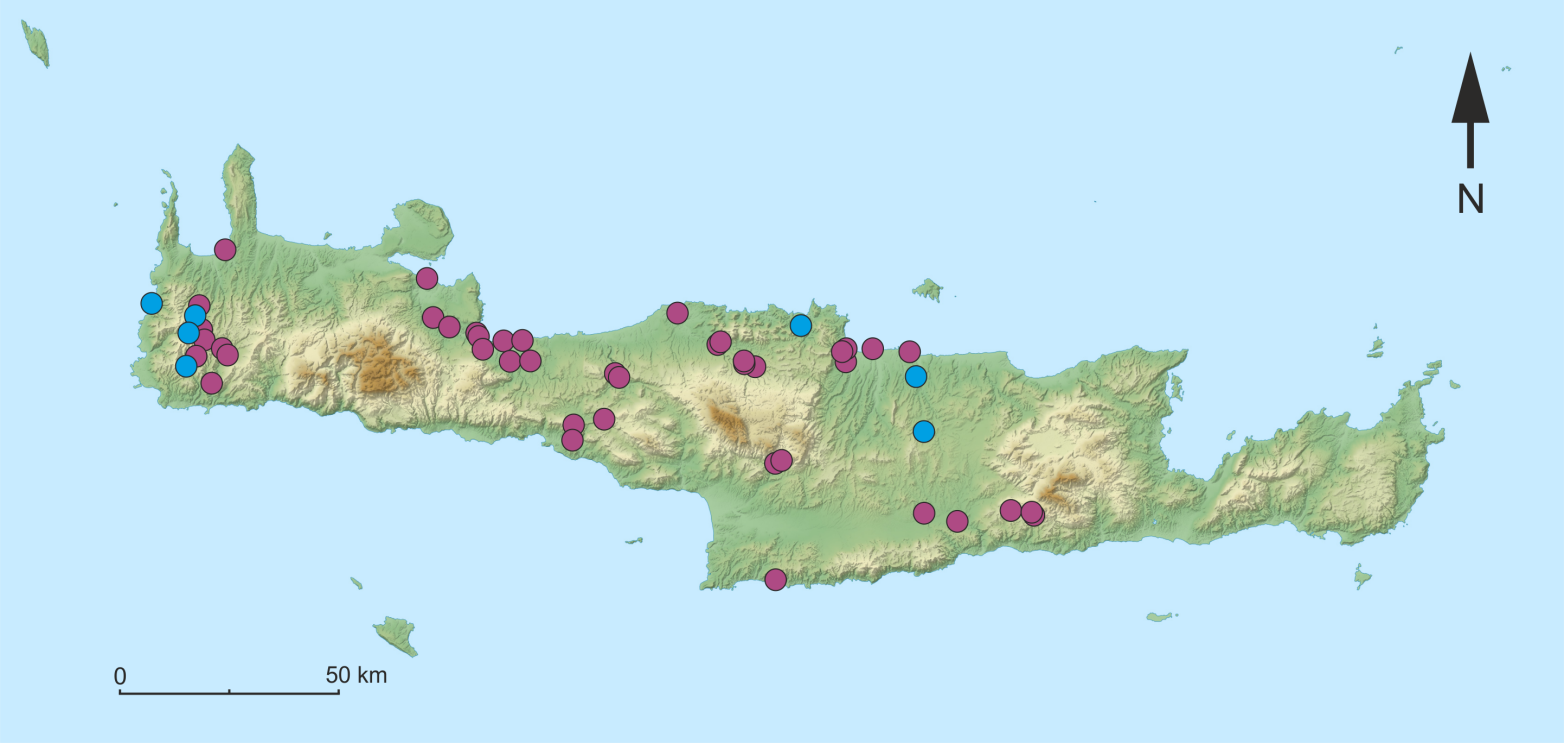
Map of the sampling sites on Crete. Purple dots indicate sites, that were visited where no individuals of ***Gammarus plaitisi*** sp. nov. were found. Blue dots represent the sites where *G. plaitisi* sp. nov. specimens were found.

## Materials and Methods

### Sample collection, identification and material deposition

The study material was collected from seven out of 53 sampling sites, including springs, streams, rivers and lakes, visited during two sampling campaigns to Crete in 2011 and 2015 (Fig. 1). Multihabitat sampling was done with rectangular kick sample nets (aperture 25 × 25 cm and 0.5 mm mesh size). The samples were sorted at the site and amphipods were immediately fixed in 96% ethanol. Afterwards, the material was evaluated with a Nikon 800 stereomicroscope. Identification to species was done according to the diagnostic morphological characters described by Karaman & Pinkster (1977a), Karaman & Pinkster (1977b), Karaman & Pinkster (1987) and by Pinkster (1993). Selected adult individuals were dissected and all the appendages of diagnostic value were stained with lignin pink (Azophloxin, C_18_H_13_N_3_Na_2_O_8_S_2_) and mounted with Euparal (Carl Roth GmBH, 7356.1) on microscope slides. Afterwards they were photographed and drawn according to the protocol described by Coleman (2006) and Coleman (2009). The body length of the specimens was measured along the dorsal side of the body from the base of the first antennae to the base of the telson. All the materials other than holotypes and paratypes are deposited in the collection of the Department of Invertebrate Zoology and Hydrobiology of University of Lodz. The type material is deposited in the Museum and Institute of Zoology Polish Academy of Sciences (catalogue numbers: MIZ 1/2018/1, MIZ 1/2018/2, MIZ 1/2018/3, MIZ 1/2018/4, MIZ 1/2018/5, MIZ 1/2018/6) and Museum für Naturkunde in Berlin (catalogue number: ZMB 30868). Relevant voucher information and sequence trace files are accessible on the Barcode of Life Data Systems (BOLD; Ratnasingham & Hebert, 2007). In addition, all the sequences were deposited in GenBank (accession numbers: COI: MG784477 to MG784549; 16S: MG784344 to MG784406; 28S: MG784423 to MG784456; ITS1: MG784460 to MG784476; EF1-*α*: MG792351 to MG792367). The electronic version of this article in Portable Document Format (PDF) will represent a published work according to the International Commission on Zoological Nomenclature (ICZN), and hence the new name contained in the electronic version is effectively published under that Code from the electronic edition alone. This published work and the nomenclatural acts it contains have been registered in ZooBank, the online registration system for the ICZN. The ZooBank LSIDs (Life Science Identifiers) can be resolved and the associated information viewed through any standard web browser by appending the LSID to the prefix http://zoobank.org/. The LSID for this publication is: (urn:lsid:zoobank.org:pub:E7EA69BA-9A8E-4B44-B999-C2BA7B69AC76). The online version of this work is archived and available from the following digital repositories: PeerJ, PubMed Central and CLOCKSS.

### Scanning electrone microscope analysis

Individuals used for scanning electron microscope (SEM) analysis were critical point dried and sputter-coated with colloidal gold (10 nm). Pictures were taken with a PHENOM PRO X SEM in the Department of Invertebrate Zoology and Hydrobiology of University of Lodz. The photographs of the composition of the pores on antenna 1 and epimeral plate 2 were taken from three same-sized individuals belonging respectively to *G. plaitisi* sp. nov. and other populations of *G. pulex pulex* under four different magnifications.

### DNA extraction, PCR amplification, sequencing, haplotype diversity and sequence analysis

About 3 mm^3^ of the muscle tissue was taken out from each individual, with a sharp-edged forceps and incubated overnight at 55 °C in a 1.5-ml tube containing 200 µl of Queen’s lysis buffer with 5 µl of proteinase K (20 mg ml^−1^) (Seutin, White & Boag, 1991). Total DNA was extracted using the standard phenol/chlorophorm method (Hillis, Moritz & Mable, 1996). Air-dried DNA pellets were resuspended in 100 µl of TE buffer, pH 8.00, stored at 4 °C until amplification and finally longterm stored at −20 °C. At first, 57 individuals from 7 sampling sites were barcoded for cox I gene fragment using LCO1490/HCO2198 (Folmer et al., 1994) and LCO1490-JJ and HCO2198-JJ (Astrin & Stüben, 2011). PCR settings for amplifying COI sequences consisted of initial denaturing of 60 s at 94 °C, five cycles of 30 s at 94 °C, 90 s at 45 °C, 60 s at 72 °C, then 35 cycles of 30 s at 94 °C, 90 s at 51 °C, 60 s at 72 °C, and final 5 min extension at 72 °C (Hou, Fu & Li, 2007). The cleaning of the PCR products was done with exonuclease I (20 U mL-1, Fermentas) and alkaline phosphatase FastAP (1 U mL-1, Fermentas) treatment according to the manufacturer’s guidelines. Subsequently, the products have been sequenced using the same primers as at the amplification stage. Sequencing of the PCR products was performed using BigDye terminator technology by Macrogen Inc.

All resulting sequences were verified and confirmed as *Gammarus* DNA via BLASTn searches in GenBank (Altschul et al., 1990) and then assembled and aligned in Geneious software (Kearse et al., 2012). The alignment was performed using MAFFT plugin with G-INS-i algorithm in Geneious software (Katoh et al., 2002).

The DNAsp software (Librado & Rozas, 2009) was used to define the haplotypes and to calculate the haplotype and nucleotide diversity. The intraspecific pairwise genetic distances were calculated in MEGA7 software (Kumar, Stecher & Tamura, 2016). The relationships between haplotypes were illustrated with median-joining network (Bandelt, Forster & Röhl, 1999) in PopArt (Leigh & Bryant, 2015).

Additional COI sequences of closely related lineages from Greece and Sweden (geographically nearest to type locality of *G. pulex pulex*), and outgroup *Gammarus* species were downloaded from NCBI GenBank and added to analyses to test the monophyly of *G*. cf *pulex* group (Table 1). The neighbour-joining tree of all COI sequences, using Tamura-Nei model of evolution with 1,000 bootstrap replicates, was created in MEGA7 software (Kumar, Stecher & Tamura, 2016).

**Table 1:** Material of *Gammarus* cf. *pulex* and outgroups used in our study.

MOTU	Locality	N	Accession number	Reference
*G. pulex pulex*	Sweden, Uppsala	1	JF965943	Hou et al. (2011)
*G. pulex pulex*	Sweden	1	JF965939	Hou et al. (2011)
*G.* cf *pulex* Greece 1	Northern Greece	2	KJ462741	Wysocka et al. (2014)
*G.* cf *pulex* Greece 2			KJ462768	
*G.* cf *pulex* Peloponnese 1	Northern Peloponnese	5	MG784489	This study
*G.* cf *pulex* Peloponnese 2			MG784478	This study
			MG784486	This study
			MG784481	This study
			MG784485	This study
*G. fossarum*	Germany: North Rhine-Westphalia	1	KT075259	Grabner et al. (2015)
*G. lacustris*	Finland: Jaekaelaevuoma	1	KX283246	Alther, Fišer & Altermatt (2016)
*G. alpinus*	Switzerland: Lai da Palpuogna	1	KX283242	Alther, Fišer & Altermatt (2016)
*G. balcanicus*	Montenegro	1	KU056219	Mamos et al. (2016)
*G. roeselii*	Albania: Lake Shkodra	1	KP789697	Grabowski et al. (2017a)

Afterwards, at least three individuals per each delimited cluster were amplified for one additional mitochondrial and two nuclear markers for phylogeny reconstruction: (1) mitochondrial 16S rRNA using 16STf and 16SBr markers (Palumbi et al., 1991; MacDonald IIII, Yampolsky & Duffy, 2005) under the following PCR conditions: initial denaturation at 94 °C for 150 s; 36 cycles of denaturation at 94 °C for 40 s, annealing at 54 °C for 40 s, extension at 65 °C for 80 s; and a final extension at 65 °C for 8 min (Weiss et al., 2014); (2) the nuclear 28S rRNA gene amplified with 28F and 28R primers (Hou, Fu & Li, 2007) under following conditions: initial denaturation at 94 °C for 3 min, 35 cycles of denaturation at 94 °C for 20 s, annealing at 55 °C for 45 s, and elongation at 65 °C for 60 s, followed by a final extension for 2 min at 65 °C and 5 min extension at 72 °C; (3) the nuclear ITS1 gene with ITS1F and ITS1R primers (Chu, Li & Ho, 2001) under following PCR conditions: 90 s at 94 °C, 33 cycles of 20 s at 94 °C, 30 s at 56.8 °C, and 30 s at 72 °C, and finally 5 min at 72 °C and EF1-*α* gene using EF1a-F and EF1a-R primers (Hou et al., 2011) under following PCR conditions: 60 s at 94 °C, followed by 35 cycles of 30 s at 94 °C, 45 s at 45–50 °C, 60 s at 72 °C, and 5 min extension at 72 °C. The nuclear markers were sequenced in both directions.

### MOTU delimitation—cryptic diversity

The Molecular Operational Taxonomic Units (MOTUs) were delimited, based on the COI marker, with five methods and two different approaches (as done before by Grabowski, Wysocka & Mamos, 2017): the distance-based approaches, namely Barcode Index Number (BIN) System (Ratnasingham & Hebert, 2013) and barcode gap discovery with the ABGD software (Puillandre et al., 2012) and the tree-based approaches, using two GMYC model-based methods (Pons et al., 2006) according to Monaghan et al. (2009) and the bPTP procedure described by Zhang et al. (2013).

The BIN method is a distance-based approach, embedded in the Barcode of Life Data systems (BOLD; Ratnasingham & Hebert, 2007). The sequences already deposited in BOLD database are confronted with the newly submitted ones. Afterwards, according to their molecular divergence, the sequences are clustered using algorithms that identify discontinuities between the clusters. A unique and specific Barcode Index Number (BIN) is assigned to each cluster. If the submitted sequences do not group together with already known BINs, a new number is created. Each BIN is registered in the BOLD database.

The Automated Barcode Gap Discovery (ABGD) method uses pairwise distance measures. ABGD clusters the sequences into MOTUs (Molecular Operational Taxonomic Units), in the way that the genetic distance between two sequences belonging to two separate groups will always be greater than an indicated threshold (i.e., barcode gap). In our study, the primary partitions were used as a principal for cluster delimitation, as they tend to remain stable on a wider range of prior values, minimising the oversplitting of the number of groups and are usually the closest to the number of taxa described by taxonomists (Puillandre et al., 2012). The default value of 0.001 was applied as the minimum intraspecific distance. As the maximum intraspecific distance we investigated a set of values up to 0.1, which has been proposed as suggested maximum distance value in amphipods distinguishing two separate species (Costa et al., 2007). The standard Kimura two-parameter (K2P) model correction was used (Hebert, Cywinska & Ball, 2003).

The bPTP approach for species delimitation is a tree based method, utilising non-ultrametric phylogenies. The number of substitutions in incorporated into the model of speciation and the bPTP assumes that the probability that a substitution leads to a speciation event follows a Poisson distribution, as the lengths of the branches of the input tree are generated independently according to either to speciation or coalescence, which are two classes of the Poisson processes. In bPTP, the Bayesian support values are added for each delimited cluster (Zhang et al., 2013). As an input tree, the phylogeny was generated using Bayesian inference in Geneious software package using MrBayes plugin (Kearse et al., 2012) with MCMC chain 1 million iterations long, sampled every 2,000 iterations. The TN93 + I + G was chosen as the substitution model, as bestfit based on bModel test (Bouckaert & Drummond, 2017). The consensus tree was constructed after removal of 25% burn-in phase. The analysis itself was done using the bPTP web server (http://species.h-its.org/) with 500,000 iterations of MCMC and 10% burn-in.

The GMYC method identifies the transition from intraspecific branching patterns (coalescent) to typical interspecific branching patterns (Yule processes) on an ultrametric, phylogenetic tree, using the maximum likelihood approach. The estimation of the boundary between coalescent and Yule branching processes can be done using two different GMYC approaches, one using the single threshold and the second one based on multiple threshold model. We have reconstructed an ultrametric tree, which is required for GMYC analyses, in BEAST software, using 20 million iterations long MCMC chain, with TN93+ I + G as the best-fit substitution model. The consensus tree was analysed in the GMYC web server (available at: http://species.h-its.org/gmyc/) using both the single and multiple threshold models.

### Time calibration and phylogeny reconstruction

The time-callibrated phylogeny was reconstructed based on data from sequences of COI (586 bp), 16S rRNA (299 bp), 28S rRNA (781 bp), ITS1 (548 bp) and EF1-alpha (602 bp) in BEAST2 software package (Bouckaert et al., 2014) with the use of five MCMC chains of 50,000,000 runs with following models of substitution: TN93 + I + G (for COI), HKY + I + G (for 16S), TN93 + I + G (for 28S), HKY + I + G (for ITS1) and TN93 + I + G (for EF1-alpha). The models for each marker were selected according to bModel test (Bouckaert & Drummond, 2017). The relaxed log-normal clock model was used and based on the selected rate of 0.0115 substitutions (SD 0.0026) per million years for COI according to already established rate (Brower, 1994), which was cross-validated against two other rates (0.0113, 0.0127) established recently for other freshwater members of *Gammarus*, in the *G. roeselii* species complex (Grabowski et al., 2017). All other clock rates were set on estimate. For 16S rRNA and EF1-alpha also relaxed log-normal clock was used, whereas for 28S rRNA and ITS1 the strict clock was used. All the models were tested beforehand in MEGA software, using an implemented test for molecular clock model based on Maximum Likelihood phylogeny (Kumar, Stecher & Tamura, 2016). The resulting trees were checked for ESS values in Tracer and two trees with the best ESS values were combined in LogCombiner and annotated in TreeAnnotator. The final output tree was edited in FigTree software (http://tree.bio.ed.ac.uk/software/figtree/).

## Results

### Systematics

**Table:** 

Order: Amphipoda Latreille, 1818
Family: Gammaridae Leach, 1814
Genus: *Gammarus* Fabricius, 1775; Pinkster, 1970: 179, Karaman & Pinkster, 1977a: 3, Barnard & Barnard, 1983: 463.

Type species: *Cancer pulex* Linnaeus, 1758 [=*Gammarus pulex* (Linnaeus)] by subsequent designation of Pinkster, 1970: 177 (neotype designation).

*Gammarus plaitisi* sp. nov

(Figs. 2–6)

**Figure 2:**
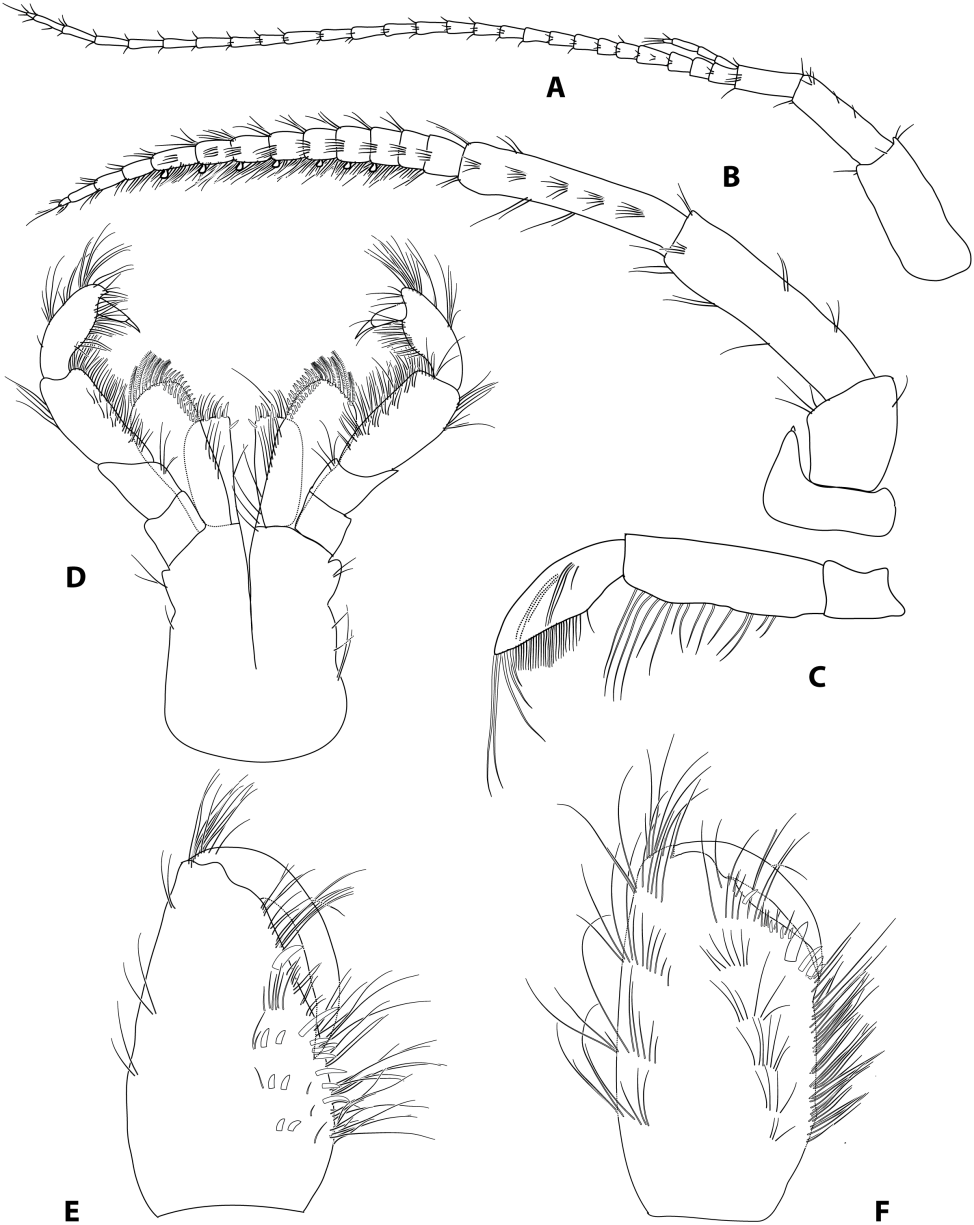
*Gammarus plaitisi* sp. nov. male, paratype, 13 mm, locus typicus, Fodele, Crete. (A) antenna 1, outer face; (B) antenna II, outer face; (C) mandibular palp, inner face; (D) maxillipeds, outer face; (E) palm of gnathopod I, outer face; (F) palm of gnathopod II, outer face.

**Figure 3:**
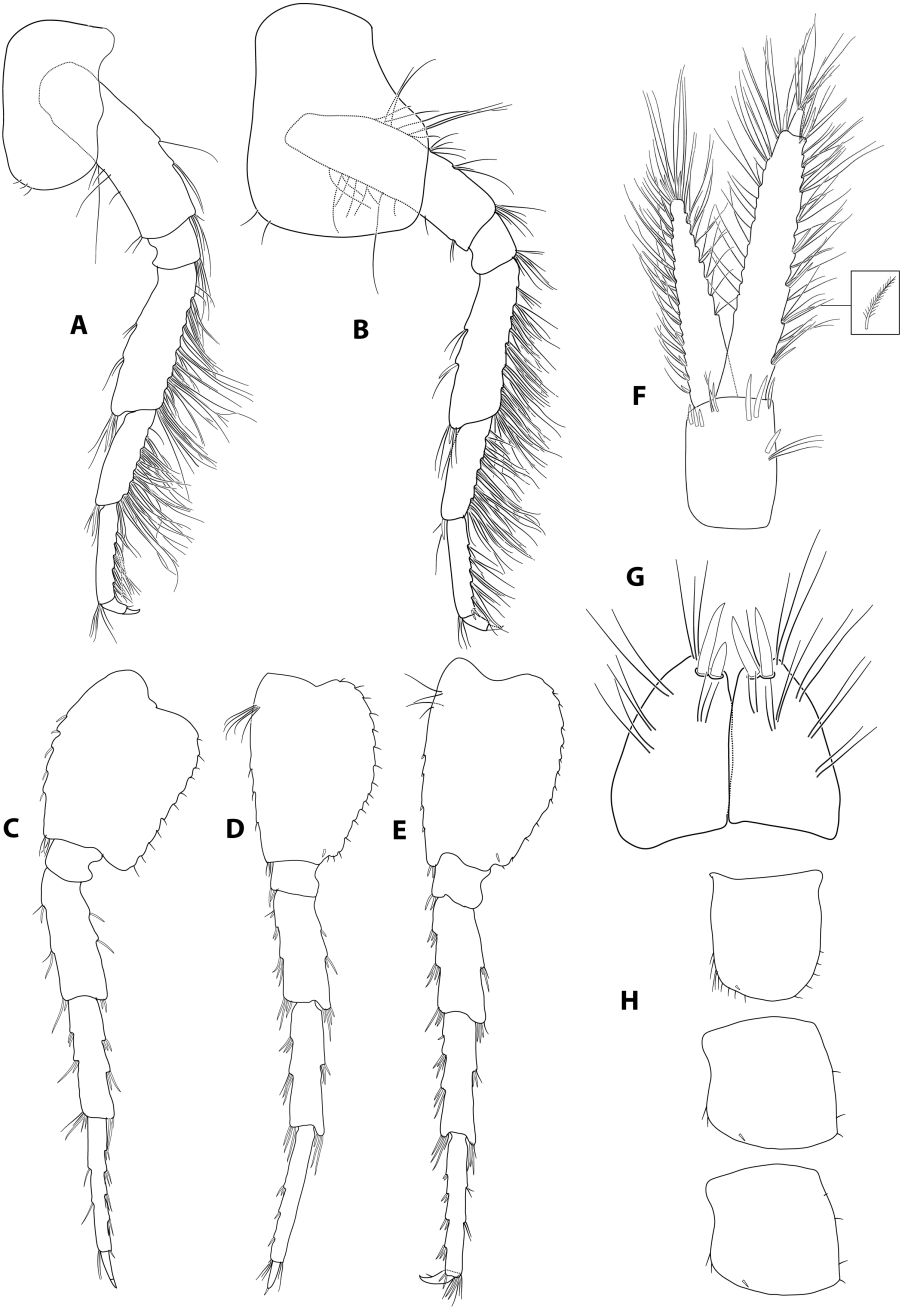
*Gammarus plaitisi* sp. nov. male, paratype, 13 mm, locus typicus, Fodele, Crete. (A–B) pereopod III and IV, outer face; (C–E) pereopod V to VII; (F) uropod III; (G) telson; (H) epimeral plates.

**Figure 4:**
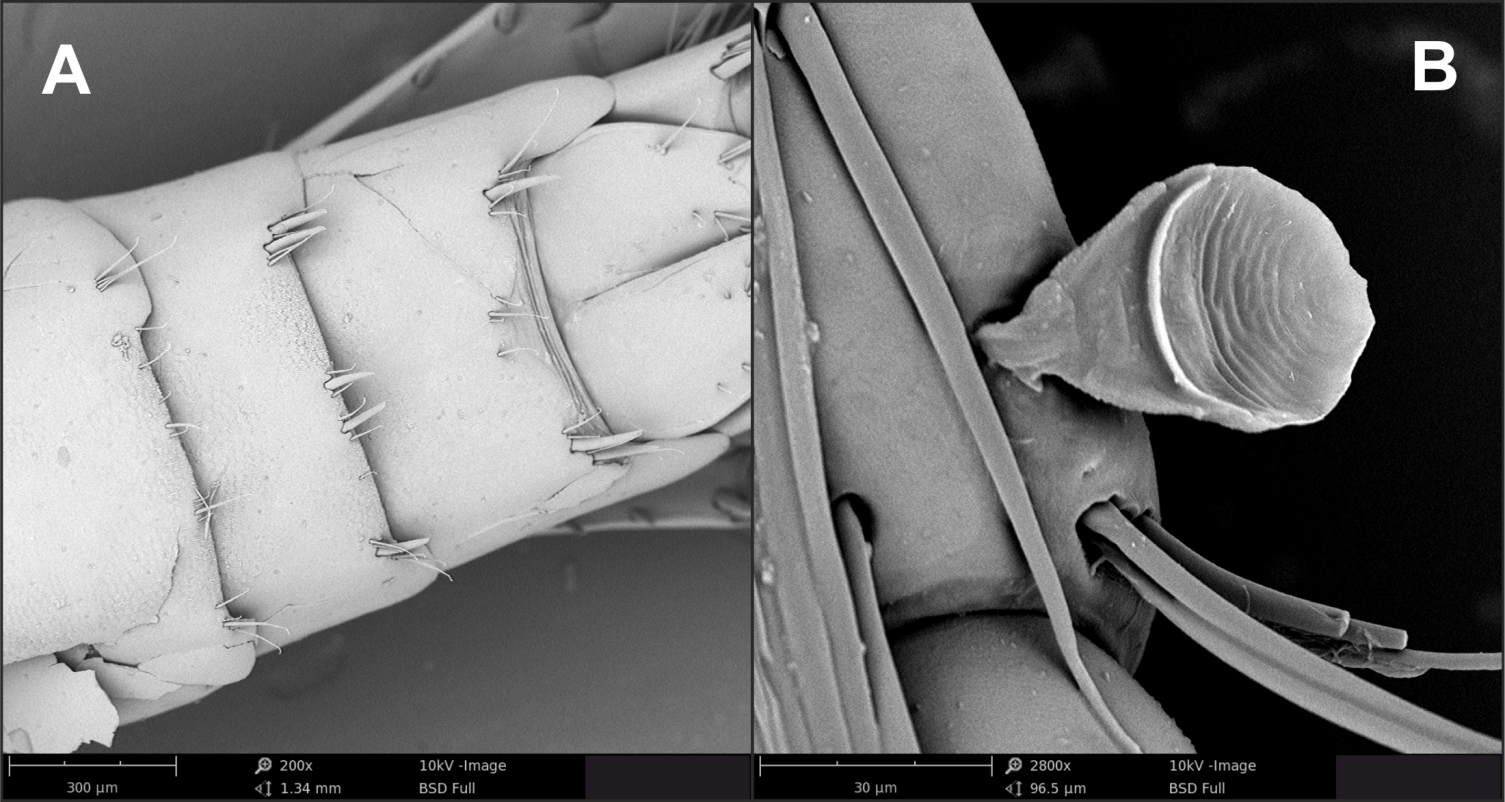
*Gammarus plaitisi* sp. nov. male, paratype, 12 mm, locus typicus, Fodele. Crete. (A) urosome, dorsal view; (B) calceola.

**Figure 5:**
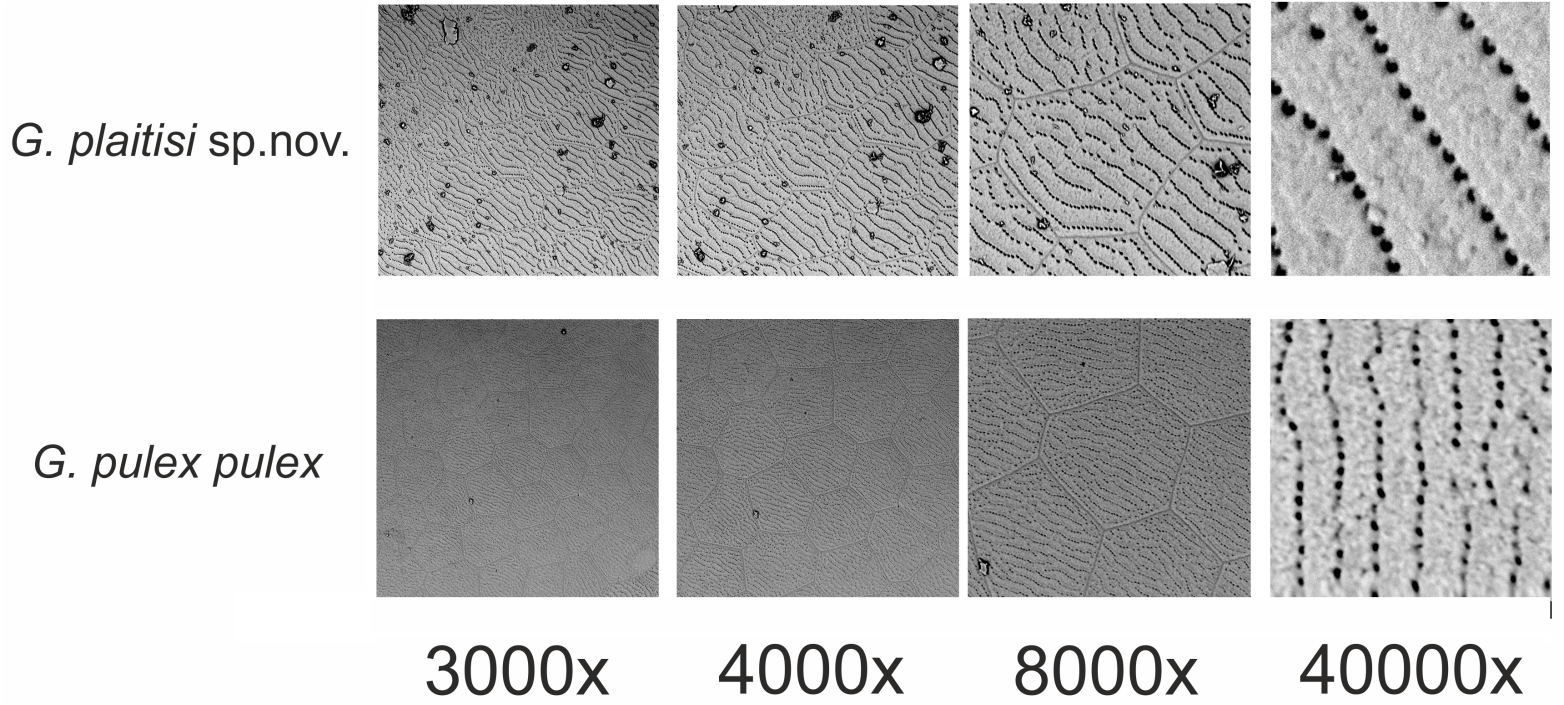
Comparison of the ultrastructure of a fragment of antenna I of *Gammarus plaitisi* sp. nov., Fodele, Crete; *Gammarus pulex pulex*, Estonia.

**Figure 6:**
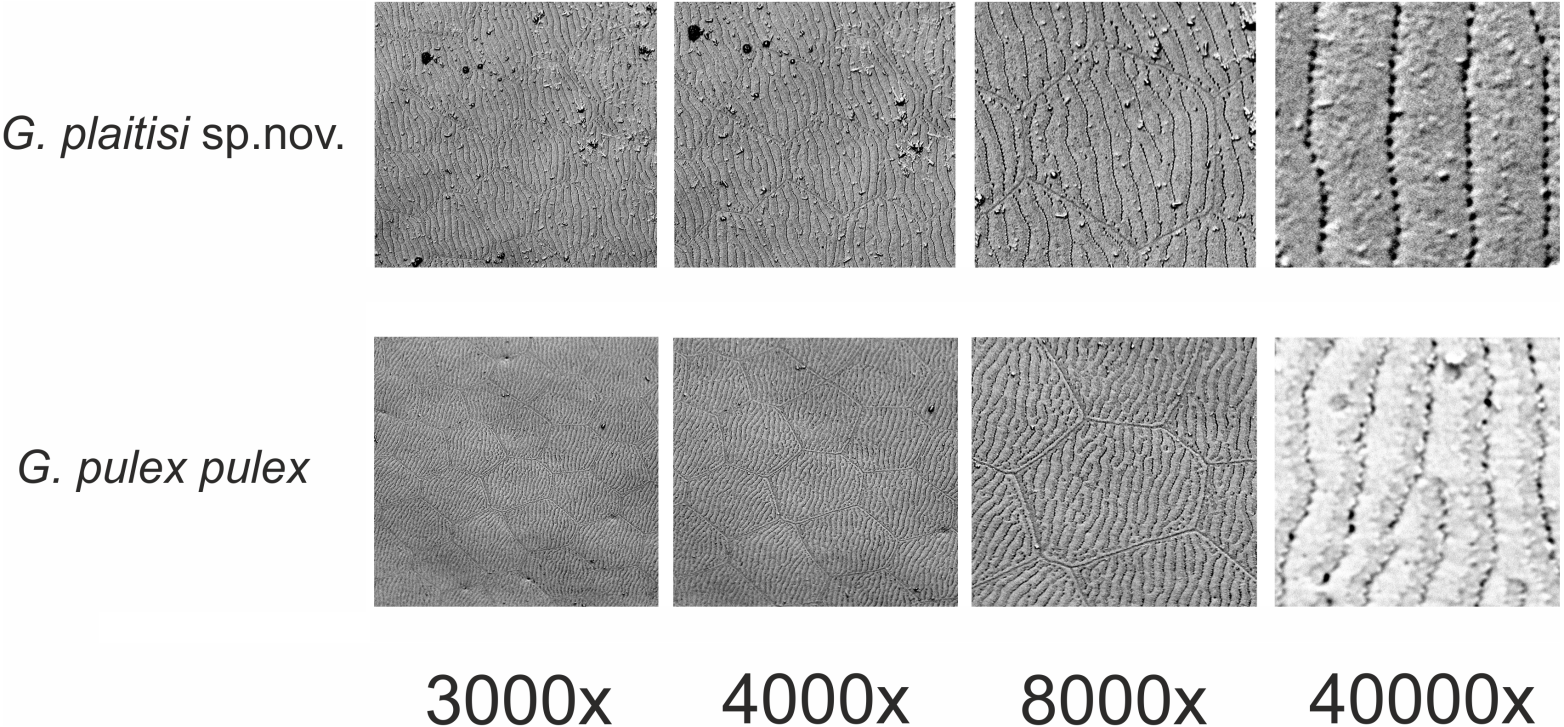
Comparison of the ultrastructure of a fragment of epimeral plate II of *Gammarus plaitisi* sp. nov., Fodele, Crete; *Gammarus pulex pulex*, Estonia.

*Gammarus pulex pulex* (part.) Karaman, 2003: 31 (Vrondisi monastery, village Zaros, Creta Island, Greece)

Diagnosis: Large species, making a robust impression. Similiar to *G. pulex pulex* by the characteristic antenna 2 with swollen flagellum, bearing a flag-like dense brush of setae and similar armature of pereiopods. It may be distinguished from *G. pulex pulex* by the lack of spines on the dorsal surface of the first segment of urosome, the shape of the posterodistal margin of the second and third epimeral plate and by the size and the arrangement of the pores on the cuticle surface. It is also clearly distinguishable from *G. pulex pulex* on the molecular level, with respect to the COI nucleotide sequence.

Materials examined: More than 200 individuals, both males and females, from 7 localities in different parts of Crete Island, Greece: *small spring and stream at the Sfinari beach* N35.41533, E23.56127, many individuals coll. 28 August 2011; *small stream in forest near Elos*, N35.36567, E23.63718, many individuals coll. 28 August 2011; *Pelekaniotikos river near Kalamios* N35.30729, E23.63583 many individuals coll. 28 August 2011; *stream near Viatos* N35.39724, E23.65512, many individuals coll. 28 August 2011; *Pantomantris River in Fodele* N35.37828, E24.95833, many individuals coll. 11 October 2015; *Springs in Astritsi* N35.19084, E25.22233, many individuals coll. 9 October 2015; *Karteros River near Skalani* N35.28893, E25.20423, many individuals coll. 9 October 2015.

Type: Holotype: An adult male individual collected on 11 October 2015, body length of 10 mm, as well as the DNA voucher (extracted DNA in buffer) deposited in Museum and Institute of Zoology Polish Academy of Sciences. Catalogue number: (MIZ 1/2018/1)); GenBank accession number: (MG784515). Paratypes deposited in Museum and Institute of Zoology Polish Academy of Sciences (catalogue numbers: MIZ 1/2018/2, MIZ 1/2018/3, MIZ 1/2018/4, MIZ 1/2018/5, MIZ 1/2018/6) and Museum für Naturkunde in Berlin (catalogue number: ZMB 30868): five specimens each fixed in 96% ethanol, collected from the type locality on 11 October 2015.

Type locality: Crete Island, Pantomantris River in Fodele, Greece. N35.37828, E24.95833

Distribution and habitat: The species is endemic to Crete. It is found in freshwaters throughout the island, usually in gravel, decomposing leaves and among submerged tree roots.

Etymology: This new species is named to honour the Cretan family Plaitis; particularly Wanda and Manolis Plaitis from Fodele village, who hosted us and provided invaluable help during our sampling expeditions to Crete.

Description: Male: Medium large, robust species with length up to 14 mm. *Head*: lateral lobes rounded; eyes small; less than twice as long as wide. *Antenna I* (Fig. 2A): about half of the body length, peduncle segments subsequently shorter with third segment about half length of the first one. Main flagellum with 25–30 segments and accessory flagellum with 3–4 segments. Both peduncle and flagellum with few short simple setae, rarely exceeding the diameter of segments. *Antenna II* (Figs. 2B and 4B): Always shorter than antenna I. Peduncle segments armed with tufts of short setae. Flagellum with 13 to 17 segments, which are swollen and compressed in adult individuals; most segments armed with transverse rows of setae on the inner surface, altogether forming a flag-like brush. Calceoli always present. *Mandibular palp* (Fig. 2C): First segment unarmed. Second segment with ventral setae: in the proximal part 2–3 setae much shorter than the diameter of the segment, in the distal part 10–13 setae as long as or up to 2.5× longer than the diameter of the segment. Third segment armed with 2 groups of long A-setae, a regular comb of 25–30 D-setae and 5–6 long E-setae. *Maxillipeds* (Fig. 2D): The maxillipeds with the inner plate armed distally with strong spine-teeth; the outer plate with spine-teeth and long plumose setae; the palp is well developed. *Gnathopod I* (Fig. 2E): Palm oblique, setose, with one strong medial palmar spine, strong angle spine accompanied by several small spines intermixed with longer setae along the posterior palmar margin with addition of small spines and short setae on the lateral surface. *Gnathopod II* (Fig. 2F): Propodus trapezoid, widening distally. Palm concave, setose, with one medial palmar spine and three angle spines. Many groups of setae, variable in length, are visible both on the inner and outer as well as the lateral surface of the propodus *Pereopod III* (Fig. 3A): Anterior and distal margin of coxal plate slightly convex, posterior margin straight. Distal corners rounded. The last three segments of third pereiopod bear groups of long, often curved setae along the posterior margin, usually two to three times longer than the diameter of segments. The anterior margin of merus armed with 1 spine. Dactylus short, robust with one seta at joint of unguis. *Pereopod IV* (Fig. 3B): Coxal plate dilated distally. Distal corners rounded. The last three segments of fourth pereiopod bear groups of long, often curved setae along the posterior margin, usually two to three times longer than the diameter of segments. The anterior margin of merus armed with 1 spine. Dactylus short, robust with one seta at joint of unguis. *Pereopod V* (Fig. 3C): Basis with a subrectangular shape, posterior margin slightly concave, posterodistal lobe well developed, posterior margin with 10–12 very short setae, anterior margin with 4–5 spiniform setae. Ischium naked. Merus, carpus and propodus with robust spines on both margins, occasionally intermixed with relatively short setae. Dactylus short, robust usually with one seta at joint of unguis. *Pereopod VI* (Fig. 3D): Similar to PV, but slightly longer and wider, posterior margin convex, posterodistal lobe less prominent and basis more more elongated with a single, little spine on posterointerior corner. Ischium to propodus armed with robust spines and very few short setae. Dactylus short, robust with one seta at joint of unguis. *Pereopod VII* (Fig. 3E): Basis wider than in PVI with a single, little spine only at posteroinferior corner and even more elongated. Further articles armed same as in preceding pereopods. *Uropod III* (Fig. 3F): The inner ramus attains about 2/3 of the length of the outer ramus. Most of setae along the inner and outer margin of endo- and exopodite plumose. *Telson* (Fig. 3G): Deeply cleft, rather setose. Each lobe with two apical strong spines intermixed with few short and long setae, several short subapical setae present. *Epimeral plates* (Fig. 3H): First epimeral plate with one spine at the laterodistal margin. Second epimeral plate with one spine at the laterodistal surface, posterodistal margin rounded. Third epimeral plate with three spines at the laterodistal surface, posterodistal margin rounded with the posterodistal corner slightly pointed. *Urosome* (Fig. 4A): very flat without any elevation. First urosomite lacking any spines on dorsomedial or dorsolateral surface and armed only with a few groups of setae. Second urosomite with dorsomedial and dorsolateral groups of robust spines (2–2–2). Third urosomite only with two groups of dorsolateral spines on each side (3–0–3), and a dorsmedial group of 2–4 setae. *Ultrastructure* (Figs. 5 and 6) The pores are larger and more distinctly marked in comparison to *G. pulex pulex*. This pattern holds true for both A1 and E2, however on A1 the difference is more pronounced. On A1 pores form the regular rows for both *G. plaitisi* sp.nov. and *G. pulex pulex*, whereas on E2 the rows of pores are much more regular in *G. plaitisi* sp.nov. compared to those in *G. pulex pulex*. The distances between rows of pores are always about 1.5 times wider than in *G. pulex pulex*. Female: Smaller than male. The setation of the peduncle segments of the first and second antennae is longer than in the male. The characteristic brush of second antenna flagellum is absent. The propodi of the gnathopods smaller than in males and the setation of P3 and P4 is less abundant and shorter.

Variability: Morphology of *G. plaiti* is stable with respect to features such as presence of calceoli in males, presence of brush in peduncle of A2, flatness and armature of urosomites. Larger individuals tend to have higher number of flagellum segments in antenna I and II, as well as more and longer setae on all appendages. The density of the setation and spinulation is also rather variable depending on age of the individual. Such variability is typical for most species of this genus (Karaman & Pinkster, 1977a; Karaman & Pinkster, 1977b; Karaman & Pinkster, 1987).

### Haplotype diversity and phylogeny reconstruction

**Figure 7:**
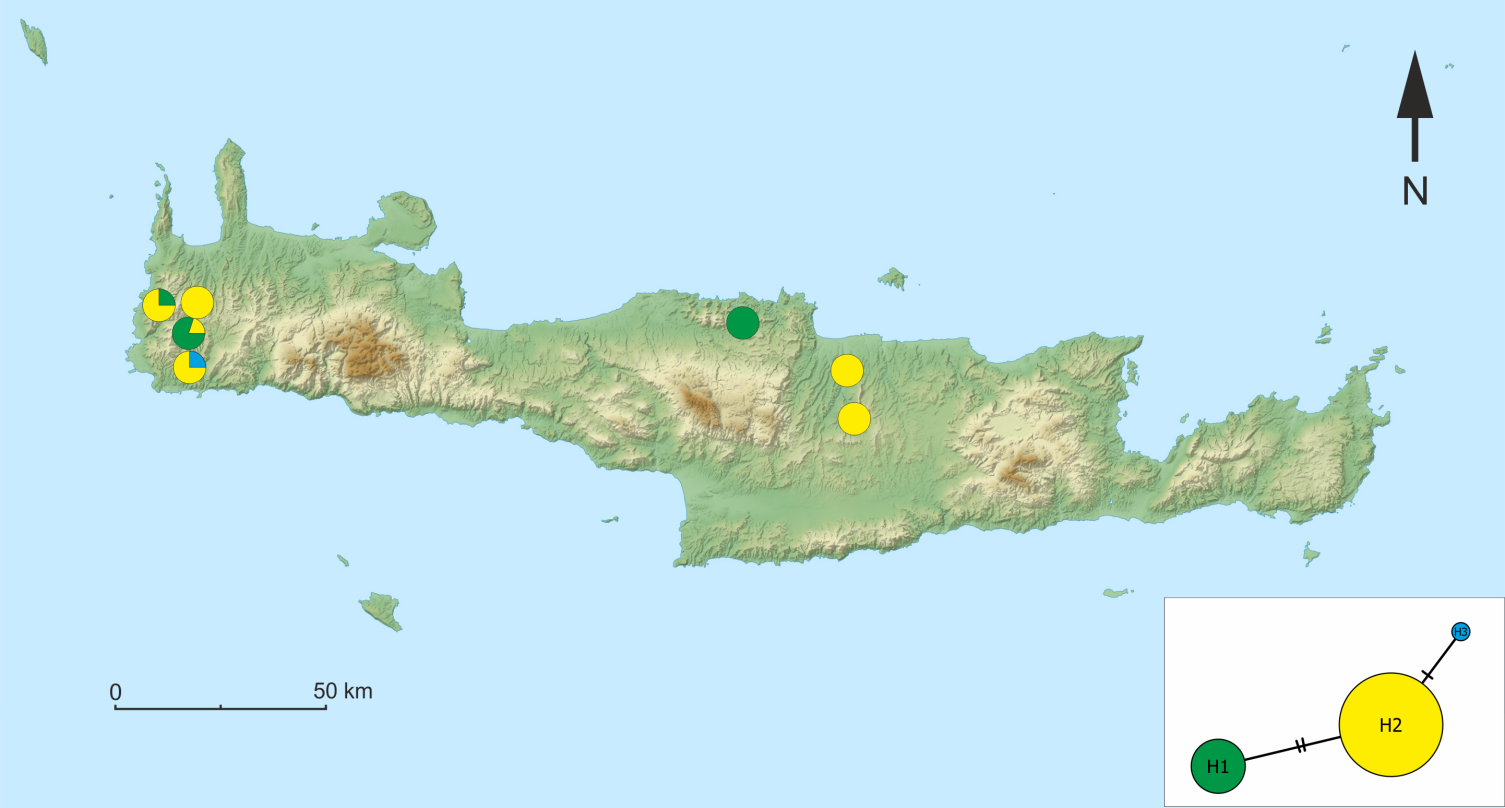
Map of the sampling sites on Crete with the median-joining haplotype network of *Gammarus plaitisi* sp. nov. Circles indicate the frequency of haplotypes at each particular site.

We identified three haplotypes of *G. plaitisi* sp.nov. in the dataset composed of the forty three COI sequences, with one haplotype being represented only by one specimen. The most common haplotype, H2, was present in the majority of sites, except for locus typicus of the species (Fig. 7). The overall haplotype diversity was quite high (Hd = 0,375 ± 0,076), whereas nucleotide diversity (Pi = 0,00126 ± 0,00075) was low. Generally, the differentiation was very low as the most common haplotype differed from the two remaining ones by a maximum of two mutation steps with intraspecific distance not exceeding the value of 0.005.

**Figure 8:**
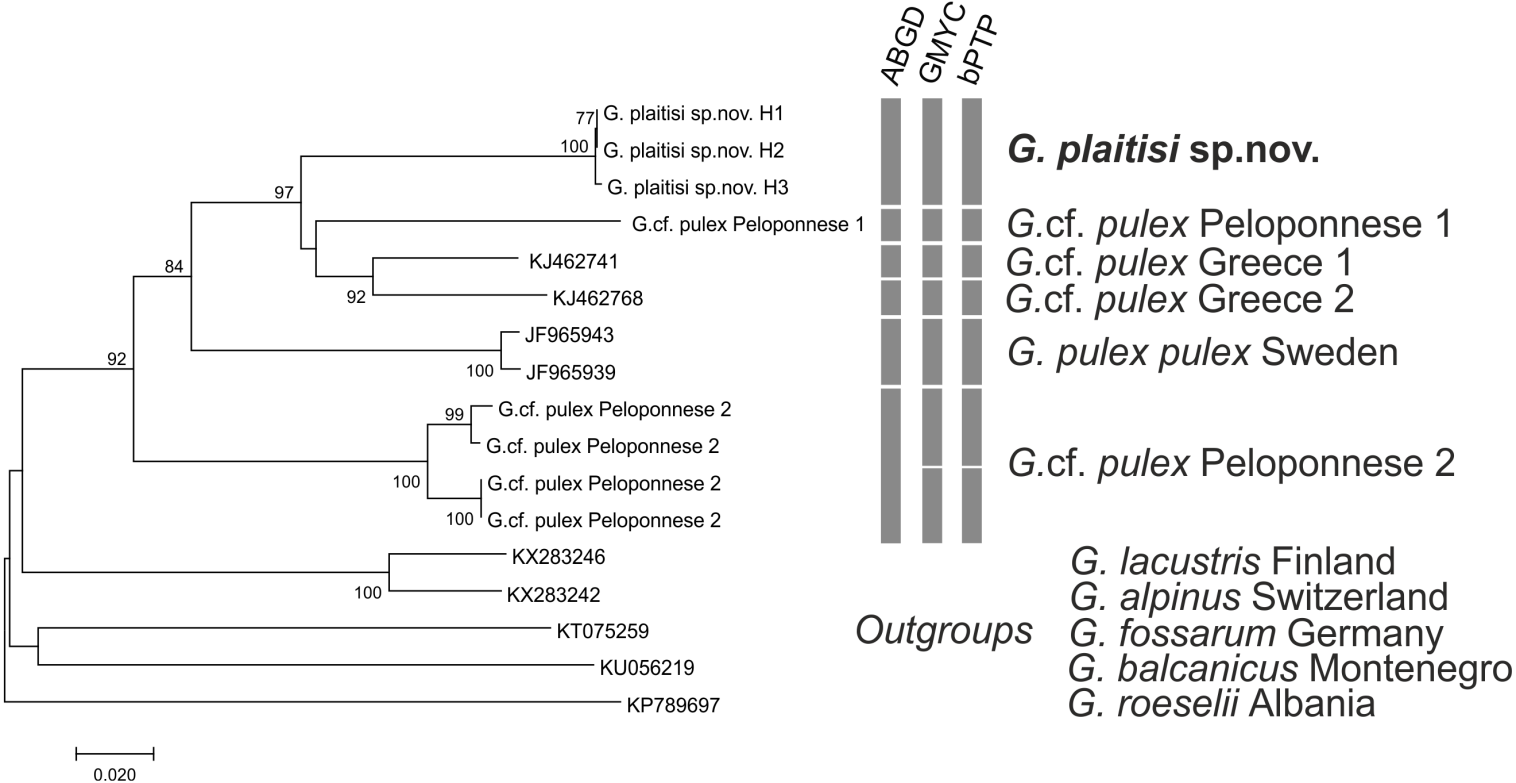
Neighbor-joining tree of the *Gammarus plaitisi* sp. nov. with members of *Gammarus* cf. *pulex*, obtained from our data and mined from NCBI GenBank with the addition of the outgroups. The numbers by respective nodes indicate bootstrap values ≥0.75. The scale bar corresponds to the number of substitutions per site. The rows of respective bars represent the delimitation of molecular operational taxonomic units (MOTU) by various methods of species delimitation.

All MOTU delimitation methods supported distinctness of *G. plaitisi*, which always formed a single MOTU and was separated from its closest relative by the mean K2P distance of 0.12 (Table S1). It also formed a unique BIN in the BOLD database (BOLD: ADG8205). All the applied MOTU delimitation methods provided constant results with six MOTUs delimited for the *G. pulex* morphospecies. Only the ABGD method indicated one MOTU less within the Peloponnese group. Both the used GMYC approaches produced the same outcome with the same LR test values. Results of MOTU delimitation methods support high cryptic diversity within *Gammarus pulex* morphospecies from Greece, as no morphological differences amongst the representatives of respective MOTUs have been found. The topology of the neighbour-joining tree confirms that *G. plaitisi* sp. nov. is nested within the clade of lineages belonging to the *G. pulex* morphospecies (Fig. 8). This suggests that *G. pulex* is, in reality, a paraphyletic group of cryptic and pseudocryptic species.

**Figure 9:**
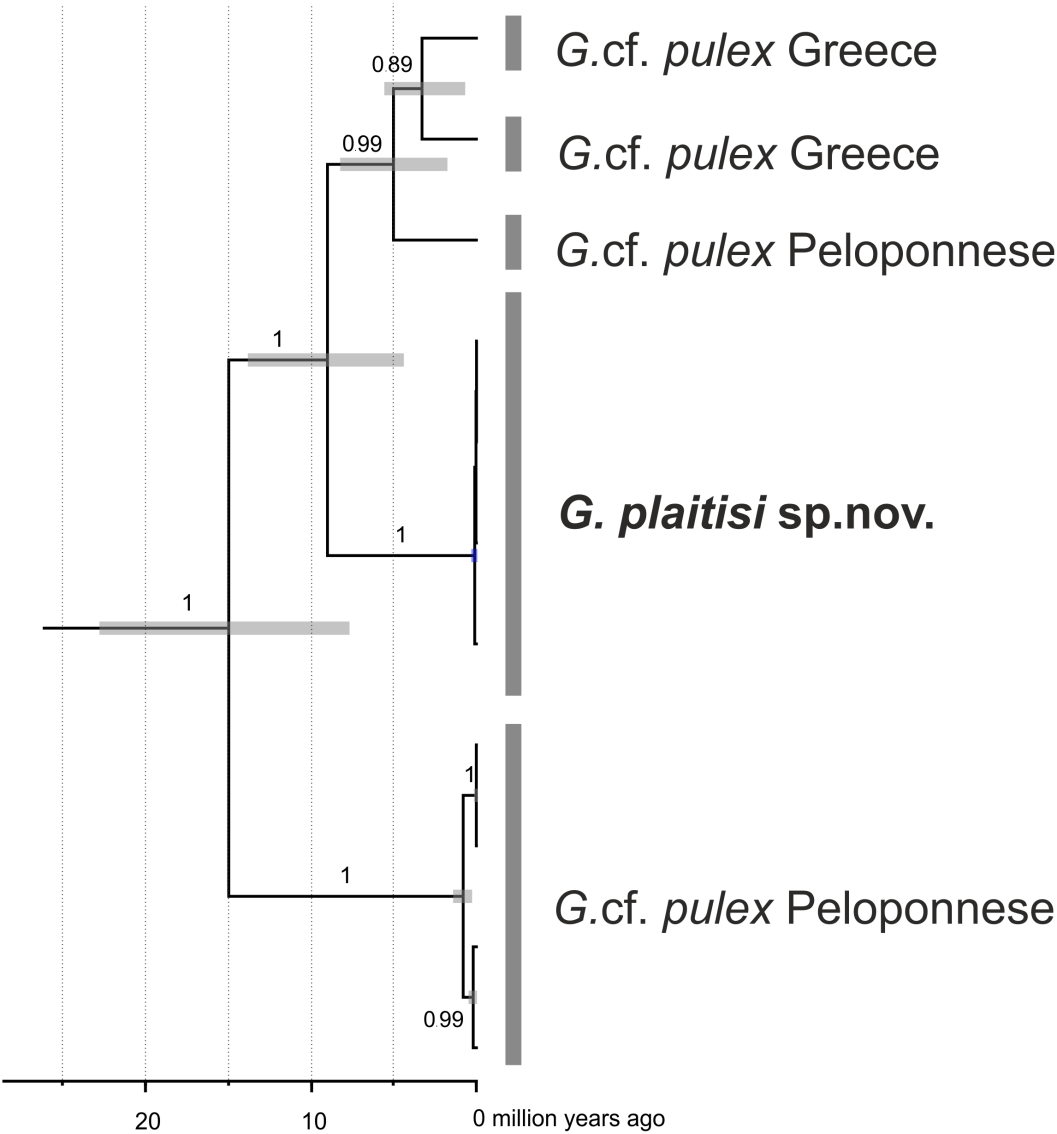
Maximum clade credibility, time-calibrated Bayesian reconstruction of phylogeny of the *Gammarus plaitisi* sp. nov. with members of *Gammarus* cf. *pulex* from Peloponnese and Northern Greece. Phylogeny was inferred from sequences of the mitochondrial COI and 16S genes and nuclear 28S, ITS1 and EF1- *α* genes. The numbers by respective nodes indicate Bayesian posterior probability values ≥0.85. Grey bars indicate the respective MOTUs of *Gammarus* morphospecies and grey node bars represent 95% HPD.

Multimarker time-calibrated phylogeny indicated that divergence of the whole *G*. *pulex* lineages from Peloponnese happened around 15 million years ago, whereas divergence of *G. plaitisi* sp.nov. from its continental relatives took place around 9.2 million years ago Moreover, divergence within the continental groups of *G*. *pulex* lineages spanned the last 5 million years (Fig. 9). All three rates used for time calibratead reconstruction of Bayesian phylogeny gave congruent results (Table 2).

## Discussion

We provided evidence for the existence of new freshwater *Gammarus* species from Crete, making this the third known freshwater endemic gammarid to Crete. The endemic freshwater species of Gammaridae before this work were, *Echinogammarus platvoeti* and *E. kretensis* (Pinkster, 1993), making *G. plaitisi* sp. nov. the first endemic of the genus *Gammarus*. The integrative taxonomy approach confirmed the distinctness of the species not only on a morphological basis, but also on a molecular level. This study also stressed the importance of using SEM photography, which may provide additional diagnostic features that are impossible to detect on usually used optical devices (Platvoet et al., 2008).

**Table 2:** Results of cross-validation of three substitution rates used in Bayesian analyses.

Node	Rate 0.0113	Rate 0.0115	Rate 0.0129
*Gammarus plaitisi* divergence from closest *G*.cf *pulex*	9.4 [4.8–14.1]	**9.2** [**4.6**–**13.8**]	8.2 [4.1–12.8]

**Table 3:** Material of ***Gammarus plaitisi*** sp.nov used in this study.

Site	Coordinates	N	Haplotype counts
PC13 (spring in Sfinari beach)	35.41533, 23.56127	4	H2 (3), H1 (1)
PC14 (stream near Elos)	35.36567, 23.63718	5	H1 (4), H2 (1)
PC17 (Pelekaniotikos river)	35.30729, 23.63583	4	H2 (3), H3 (1)
PC22 (spring near Vlatos)	35.39724, 23.65512	4	H2 (4)
KPM22 (springs in Nikos Kazantzakis)	35.19084, 25.22233	15	H2 (15)
KPM23 (stream 8km from Iraklion)	35,28893, 25,20423	7	H2 (7)
KPM33 (Fodele, locus typicus)	35.37828, 24.95833	4	H1 (4)

Despite the presence of *G. plaitisi* sp. nov. in seven, mostly isolated sites located both in the eastern and western part of Crete, its haplotype diversity is surprisingly low, with only two mutation steps separating the three known haplotypes (Table 3). This pattern suggests a strong founder effect and recent dispersal, probably in the late Pleistocene, as suggested by the time-calibrated phylogeny, possibly due to rearrangement of the local hydrological networks at the end of the last ice age. This is a rather unusual finding considering the fact that Pleistocene glaciations, which strongly affected the river systems, promoted the diversification of various taxa in the Mediterranean (Previšić et al., 2009; Goncalves et al., 2015), including the freshwater gammarids (Grabowski et al., 2017a). However, such a founder effect scenario has also been found in other freshwater members of the genus *Gammarus*, such as *Gammarus minus* which inhabits both surface and groundwaters of North America. Gooch & Glazier (1986) confirmed postglacial dispersal of this species from refugia, which resulted in strong decrease in their allele diversity. This scenario is the most plausible one also for *G. plaitisi* sp. nov., which may have colonised the current distribution area from a single refugium. The distribution of haplotypes (Fig. 7) suggests that the individuals originate from a founding population from the western part of Crete, where all of the known haplotypes are present. Yet another question concerns the way of dispersal between isolated freshwater systems, separated by more than 100 km. One must consider passive dispersal i.e., by birds (Rachalewski et al., 2013), however, groundwater connections cannot be excluded (Harris, Roosa & Norment, 2002). On the other side, there may be still some localities, particularly in the mountains, where the species is present or could have been present in the early Holocene but died out due to climatic changes. We still do not have enough data to reveal the dispersal history of this species.

Our results suggest that *G. plaitisi* sp. nov. has diverged from the continental lineages of *G*. *pulex* around 9 million years ago (Fig. 9). This result has been strongly supported by cross-validation with other substitution rates proposed for freshwater gammarids in earlier studies (Grabowski et al., 2017a). The timescale seems to be convergent with the estimated date of the first isolation of Crete from Peloponnese (Poulakakis et al., 2015). Since that time Crete could be colonized only by overseas dispersal. This finding suggests the continental origin of the newly described species. The molecular data suggest rather the possibility of its dispersal to Crete before first isolation of this island than migration during the temporal land connection during the Messinian Sality Crysis and after its final isolation at around 5 million years ago.

The closest known relatives to *G. plaitisi* sp.nov. are continental lineages of *G*. *pulex* from Peloponnese and the northern Greece (Fig. 8). These continental lineages diverged from each other around 5 million years ago, during the time of the Messinian Salinity Crisis (5.96–5.33 Mya), when the Mediterranean Basin dessicated (Krijgsman et al., 1999). The reopening of the Strait of Gibraltar ended the Messinian Salinity Crisis and resulted in refilling of the basin (Hsü et al., 1977). Nesting of *G. plaitisi* sp. nov. in between lineages of *G. pulex pulex* confirms the already known lack of monophyly present in a number of freshwater gammarid morphospecies (i.e., Hou et al., 2011; Hou, Sket & Li, 2014; Weiss et al., 2014; Mamos et al., 2014; Mamos et al., 2016; Copilaş-Ciocianu & Petrusek, 2015; Copilaş-Ciocianu & Petrusek, 2017; Katouzian et al., 2016; Grabowski et al., 2017a; Grabowski, Wysocka & Mamos, 2017b). These data support the need for a comprehensive revision of *Gammarus pulex*.

## Conclusions

*G. plaitisi* sp. nov. is the first endemic insular freshwater *Gammarus* in the Mediterranean. However, given the scarcity of the sampling in the fresh waters of the Mediterranean islands, there is a high chance there are more representatives of the genus in the Aegean Basin and other Mediterranean islands. The description of this new species using the integrative taxonomy approach not only broadens the knowledge about freshwater diversity of Crete, but also provides a link between the geological history of this island with the evolution of the local freshwater species. The results provide yet another piece of the puzzle in explaining the evolution of the family Gammaridae.

##  Supplemental Information

10.7717/peerj.4457/supp-1 Figure S1 Maximum clade credibility, time-calibrated Bayesian reconstruction of phylogeny of the *Gammarus plaitisi* sp. nov. with members of *Gammarus* cf. *pulex*, including the outgroup of *Gammarus cf. balcanicus* Phylogeny was inferred from a sequences of the mitochondrial COI and 16S genes and nuclear 28S, ITS1 and EF1- *α* genes. The numbers by respective nodes indicate Bayesian posterior probability values ≥0.85. Grey bars indicate the respective MOTUs of *Gammarus* morphospecies and grey node bars represent 95% HPD.

10.7717/peerj.4457/supp-2 Table S1 The intra- and interspecific pairwise genetic distances

10.7717/peerj.4457/supp-3 Supplemental Information 1 Various DNA sequences used in analyses described in manuscript

## Additional Information and Declarations

### Data Availability

The following information was supplied regarding data availability: GenBank and BOLD databases. The DNA sequences are deposited in the BOLD dataset, which is publicly available. GenBank accession numbers: COI: MG784477 to MG784549; 16S: MG784344 to MG784406; 28S: MG784423 to MG784456; ITS1: MG784460 to MG784476; EF1-*α*: MG792351 to MG792367.

### New Species Registration

The following information was supplied regarding the registration of a newly described species: Publication LSID: urn:lsid:zoobank.org:pub:E7EA69BA-9A8E-4B44-B999- C2BA7B69AC76; *Gammarus plaitisi*: urn:lsid:zoobank.org:act:3E3B2B0F-60AD-469D-B896- A25003E3FF3B.
