# Supplementary figures and images for: First endemic freshwater Gammarus from Crete and its evolutionary history—an integrative taxonomy approach
Source: PeerJ. 2018 Mar 9;6:e4457. doi: 10.7717/peerj.4457 (PMC5846458; doi:10.7717/peerj.4457)

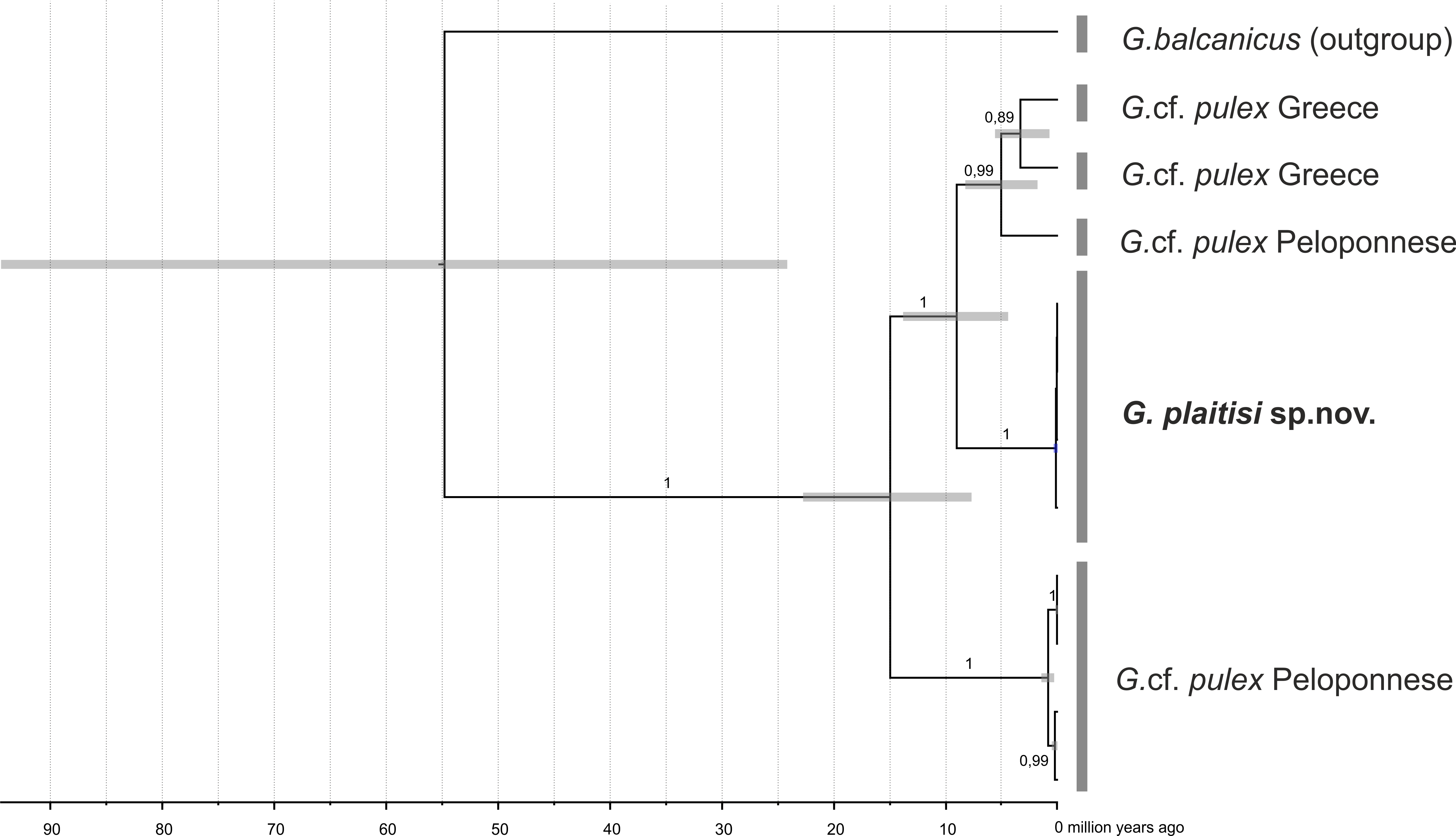

Supplement: Figure S1 — Phylogeny was inferred from a sequences of the mitochondrial COI and 16S genes and nuclear 28S, ITS1 and EF1- α genes. The numbers by respective nodes indicate Bayesian posterior probability values ≥0.85. Grey bars indicate the respective MOTUs of Gammarus morphospecies and grey node bars represent 95% HPD. [file peerj-06-4457-s001.png]
